# MuscleJ2: a rebuilding of MuscleJ with new features for high-content analysis of skeletal muscle immunofluorescence slides

**DOI:** 10.1186/s13395-023-00323-1

**Published:** 2023-08-23

**Authors:** Anne Danckaert, Aurélie Trignol, Guillaume Le Loher, Sébastien Loubens, Bart Staels, Hélène Duez, Spencer L. Shorte, Alicia Mayeuf-Louchart

**Affiliations:** 1 UTechS Photonic BioImaging/C2RT, Institut Pasteur, Université Paris Cité, 75015 Paris, France; 2 French Armed Forces Biomedical Research Institute - IRBA, Brétigny-sur-Orge, France; 3 École Centrale d’Electronique (ECE), Paris, France; 4 CHU Lille, INSERM, Institut Pasteur de Lille, Univ. Lille, U1011-EGID, Lille, 59000 France; 5 Service Neuropédiatrie, CHU Lille, 59000 Lille, France

**Keywords:** Histology, Muscle fiber morphology, Centro- and perinuclei, Fiber typing, Vascularization, Phenotype cartography, Extracellular matrix, Interstitial cells, Sarcolemmal staining

## Abstract

Histological analysis of skeletal muscle is of major interest for understanding its behavior in different pathophysiological conditions, such as the response to different environments or myopathies. In this context, many software programs have been developed to perform automated high-content analysis. We created MuscleJ, a macro that runs in ImageJ/Fiji on batches of images. MuscleJ is a multianalysis tool that initially allows the analysis of muscle fibers, capillaries, and satellite cells. Since its creation, it has been used in many studies, and we have further developed the software and added new features, which are presented in this article. We converted the macro into a Java-language plugin with an improved user interface. MuscleJ2 provides quantitative analysis of fibrosis, vascularization, and cell phenotype in whole muscle sections. It also performs analysis of the peri-myonuclei, the individual capillaries, and any staining in the muscle fibers, providing accurate quantification within regional sublocalizations of the fiber. A multicartography option allows users to visualize multiple results simultaneously. The plugin is freely available to the muscle science community.

**Supplementary Information:**

The online version contains supplementary material available at 10.1186/s13395-023-00323-1.

## Main

The histological study of skeletal muscle is an efficient way to understand its pathophysiological state, especially in the context of myopathies, aging, or responses to exercise and regeneration. Histological analysis is particularly useful in establishing a diagnosis and understanding the progression of various pathological conditions or for evaluating potential therapeutic approaches. Different parameters of skeletal muscle sections are examined to generate quantitative measurements of specific readouts. For example, the fiber cross-sectional area (CSA) and Feret diameter of muscle fibers can be used to evaluate skeletal muscle atrophy/hypertrophy. However, the amount of histological detail that can be obtained from a skeletal muscle tissue slide is large and often underexploited, mostly due to the subjectivity and massive time consumption of manual feature assessment. Therefore, several software programs (Sup. Table 1) have been developed to automate the estimation of these different parameters, including MuscleJ [1], an automated ImageJ macro that we created to quantify multiple types of histological data from muscle immunofluorescence slide images.

Initially, MuscleJ was able to automatically extract the fiber CSA and Feret diameters, the number of centronucleated fibers, the number of centronuclei, the number of satellite cells and capillaries (initially called “vessels”) per fiber, and the fiber typing [1]. MuscleJ begins its analysis with fiber segmentation, which then defines four regions of interest (ROIs): the ROI fiber (ROI^F^), the ROI centronucleated fibers (ROI^CNF^), the ROI satellite cells (ROI^SC^), and the ROI vessels (ROI^V^). Specific staining is then quantified, and the results are automatically stored in results’ files. This automated process enables the high-content analysis of raw immunofluorescence image batches. Since its development, MuscleJ has been used in many studies. However, user requests prompted us to implement additional functions, which we have bundled in a new plugin named MuscleJ2, which enables faster analysis.

Along with a new interface, additional extracted features include the quantification of peripheral myonuclei, the evaluation of vascularization, and the characterization of specific cells anywhere in skeletal muscle. We also quantify the fluorescence intensity of any immunolabeling within muscle fibers and have made it possible to analyze this staining in multiple ROIs. A fifth ROI corresponding to the region bordering the muscle fiber membrane was also added. Another new feature is the ability to quantify any staining of extracellular matrix (ECM) components, such as different types of collagen or laminin. We have improved the sensitivity of the software for different quantification workflows and added numerous new measurements to the table of results. In MuscleJ2, users can perform multioutput analyses and multicartographies to obtain a full characterization of skeletal muscle tissue. The plugin is freely available in a publicly shared space (https://github.com/ADanckaert/MuscleJ2/) and will be updated regularly.

## Results

**Fig. 1:**
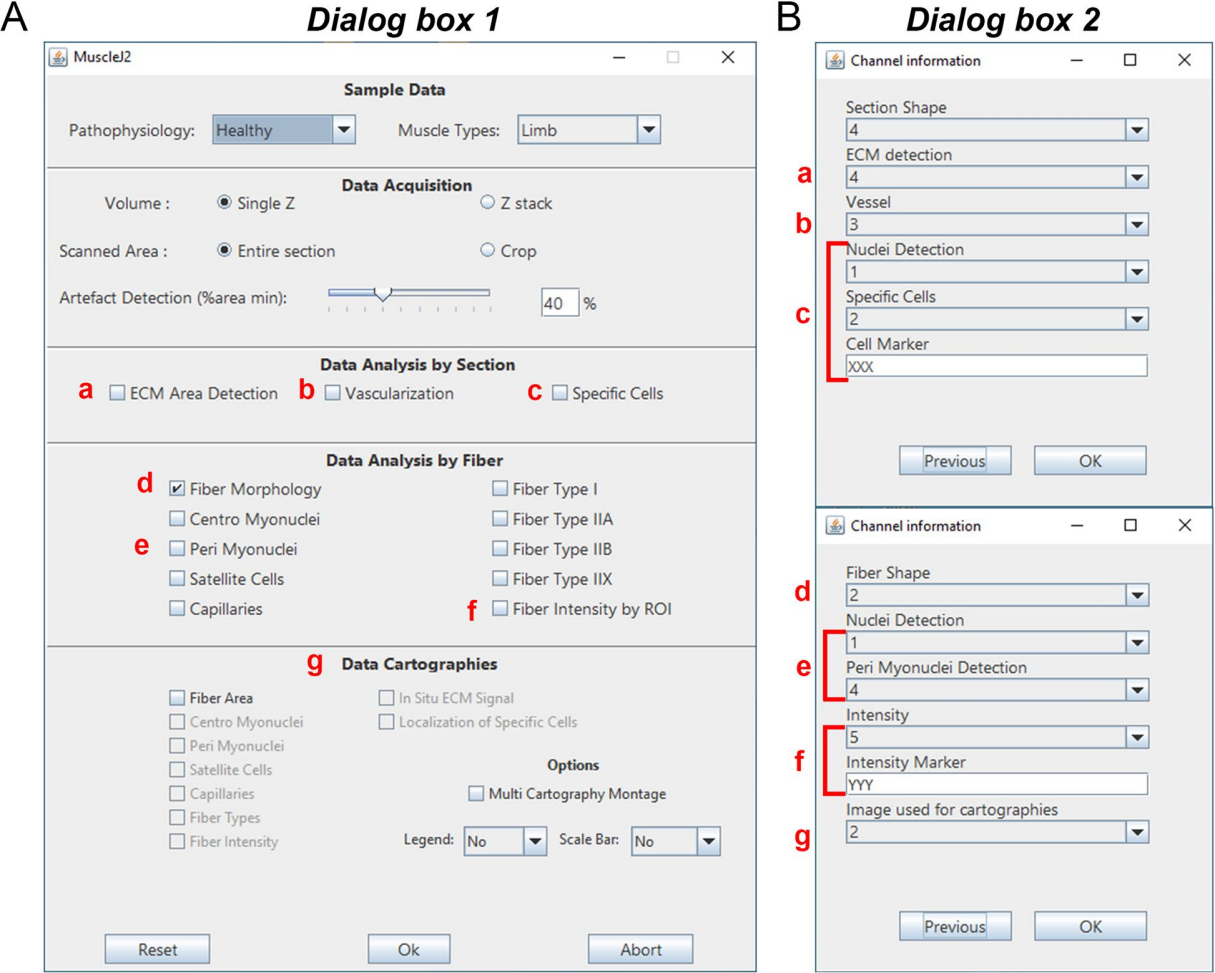
Interface of the MuscleJ2 plugin. Screenshots of the plugin dialog boxes. **A** The main dialog box MuscleJ2 is divided into five sections where the user must select from a drop-down menu or check boxes. The lowercase letters in red refer to dialog box 2, in which the channels and staining information must be indicated. **B** The *Channel information* dialog box is used to indicate the channel number for each requested analysis. Depending on the analyses selected in the MuscleJ2 dialog box, the design of this dialog box changes. In the upper panel, the lowercase letters in red refer to the section *Data Analysis by Section* (a, b, c); in the lower panel, they refer to the section *Data Analysis by Fiber* (d, e, f) and *Data Cartographies* (g)

The user interface of MuscleJ2 is organized into five panels: *Sample Data*, *Data Acquisition*, *Data Analysis by Section*, *Data Analysis by Fiber*, and *Data Cartographies* (Fig. 1), which will be described in more detail below. Before starting a run on an image set, the user must organize the acquired images into different folders so that the images in a given folder have the same properties (same type of muscle, same pathophysiological state, same staining, same data acquisition), as explained in the online *User Guide*.

### Sample data panel

Under physiological conditions, CSA is homogeneous across fiber regions, making it possible to use this parameter to discriminate between what can and cannot be labeled as a fiber. This is not the case when skeletal muscle is damaged, as in myopathies or after injury, where fiber size can be very heterogeneous. We have taken this point into account and have introduced an option, named *Pathophysiology*, where the user can choose between *Healthy* and *Damaged* fiber populations (Fig. 1). In damaged muscles, the heterogeneity of fiber CSA is increased, and MuscleJ2 flexibly considers a wider range of CSA measurements. The contribution of the *Damaged* option (selected in the *Pathophysiology* tab) is illustrated in Fig. S1, where the mouse *tibialis anterior* was partially injured, resulting in significant variability in fiber CSA between injured and uninjured parts. When *Healthy* is selected, MuscleJ2 excludes the largest and smallest fibers. When the *Damaged* option is selected, the range of differences in fiber CSA is much wider, and all the fibers are taken into account. This option allows the users to adapt the algorithm according to their parameters of interest. Notably, even in fusiform muscles such as the *tibialis anterior*, not all myofibers are fully aligned with the longitudinal axis of the muscle, and some have a high pennation angle [2]. This has led to the presence of nontransversal but extremely elongated fibers within cross sections (in parts of rat muscles) holding a low circularity value, and these fibers were correctly excluded by MuscleJ2. There can also be variation in CSA values along the length of the muscle [3], which would require the analysis of multiple levels of cross sections for a better assessment of myofiber size variation.

In the *Sample Data* panel, the user can inform the plugin of the anatomical origin of the sections, i.e., from limb or diaphragm muscle (Fig. 1). This option was added because of the large difference between classical hind limb muscles and the diaphragm, the latter usually being cut in a folded state (Fig. S2). When the *Diaphragm* option is selected, MuscleJ2 does not fill in holes to account for the actual surface of the tissue. As this type of skeletal muscle is studied with particular interest in pathological states [4, 5], this option now offers the possibility of analyzing it with MuscleJ2.

It is now possible to analyze a section of skeletal muscle divided into several pieces in the image, whereas in the first version of MuscleJ, only the largest region was selected. This allows the analysis of different skeletal muscle subsections grouped on the same image, which is particularly useful for muscles with different chiefs, such as the *quadriceps femoris* or the *gastrocnemius*, which can be separated into several parts during the cryosection preparation.

### Data acquisition panel

We have developed an algorithm applicable to images obtained from a wider range of more recent equipment, which is why the selection of the *Acquisition system* (*Apotome*/*Wide field*/*…*) and the *File format* is no longer necessary. MuscleJ2 can easily work on different image formats (such as.czi,.lif,.tiff …) supplied by the majority of gold standard image acquisition systems (Fig. S3). Importantly, image quality is a prerequisite for good analysis, and the acquisition system must be carefully selected before the batch experiments are performed.

In the *Volume* option, the user must inform MuscleJ2 if the images contain a single Z or a stack of Z. When the *Z-stack* option is selected, MuscleJ2 will automatically perform a maximum intensity projection prior to any analysis (Fig. S3). Although MuscleJ2 is designed to work on whole skeletal muscle sections, there is a *Scanned Area* option (*Entire section/Crop*) in case the muscle section is not whole. However, the user must be careful when using the *Crop* option and ensure that the crop contains a minimum of 25% of the image with a black background without tissue. This is essential for correct quantification.

The *Artefact Detection* option, which was previously in the MuscleJ macro, has been incorporated into this section (Fig. 1). It allows the user to eliminate from the analysis any slides where the detected muscle fibers represent less than the indicated percentage of the total muscle surface.

### A new panel with features related to the whole skeletal muscle section

In this third panel, named *Data Analysis by Section*, we have introduced new functionalities that do not refer to individual fibers but to the total surface of the skeletal muscle (Fig. 1). All these analyses are performed on whole-slide image sections or on representative parts of the image manually cropped by users. For these analyses, the definition of ROI is not necessary, unlike other functionalities of the *Data Analysis by Fiber* panel, described below. Consequently, laminin staining is not mandatory, and artifact detection is not associated with these options. It is therefore the responsibility of the user to ensure that the muscle sections are correctly detected and do not contain holes or folds. However, staining for ECM or any fiber marker (except nuclear markers) is necessary for MuscleJ2 to delineate the section contours and estimate the total surface area. The corresponding channel must be implemented in the *Section Shape* in dialog box 2 (Fig. 1). This allows the quantifications of the different parameters to be related to the total surface of skeletal muscle.

In this panel, three new features have been developed:

#### *ECM Area Detection (*Fig. 2A*)*

**Fig. 2:**
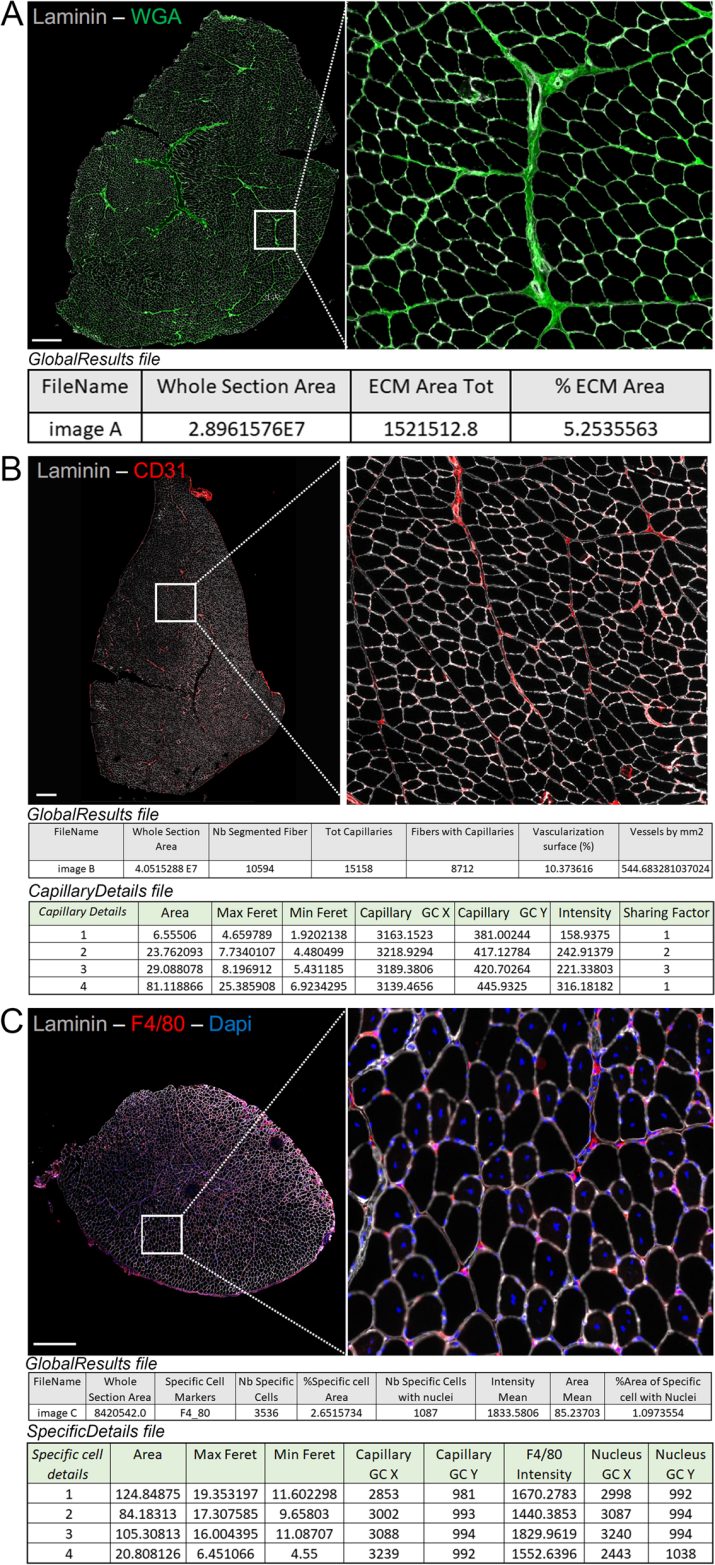
New functionalities of the plugin. **A** Immunostaining of skeletal muscle with WGA showing the extracellular matrix (ECM) in green (*SB* = 600 µm) and respective quantification with MuscleJ2 in the "GlobalResults" file. **B** Immunostaining of skeletal muscle with laminin (gray) and CD31 showing the endothelial cells in red (*SB* = 600 µm) and quantification of vessels and capillaries with MuscleJ2. Tables present the results obtained after selecting the option *Vascularization* (section *Data Analysis by Section*) and *Capillaries* (section *Data Analysis by Fibers*). The gray table presents the results obtained in the "GlobalResults" file, and the green table presents some of the results obtained in the "CapillaryDetails" file (*SB* = 600 µm). **C** Immunostaining of skeletal muscle with laminin (gray), DAPI (blue), and F4/80 showing the macrophages in red and quantification of specific cells with MuscleJ2 (*SB* = 600 µm). The gray table presents the results obtained in the "GlobalResults" file, and the green table presents some of the results obtained in the "*SpecificCells*" file. *Nucleus GC* *X* and *Y* correspond to the coordinates of identified specific cells colabeled with DAPI. All areas are indicated in µm^2^

The ECM forms a network of macromolecules and smaller components that fill the extracellular space and can be divided into two parts: the basement membrane, which surrounds thin muscle fibers, and a more diffuse interstitial matrix. The basement membrane can be specifically detected using anti-laminin or anti-collagen IV antibodies, for example. Quantification of the ECM is particularly important in the context of myopathies and skeletal muscle regeneration studies, which require assessment of the area of fibrosis corresponding to modifications of the ECM, with accumulation of different components, such as collagen I (reviewed in Loreti et al. [6]). Similarly, wheat germ agglutinin (WGA), a carbohydrate-binding protein conjugated to various fluorochromes, can be used for the global visualization of muscle ECM and fiber boundaries [7]. This provides rapid fluorescence staining with few background noise events (Fig. 2A). Since WGA detects ECM by labeling sialic acid and N-acetylglucosamine residues contained in glycoproteins and glycolipids, it could also be linked to oligosaccharides contained in the cell membrane. Therefore, we do not recommend its utilization in conditions with large and multifocal myofiber necrosis areas and/or with immune infiltrates, such as the first few days after muscle injury (data not shown).

Because ECM staining is sufficient and is included in the algorithm to detect the muscle section, another channel for the *Section Shape* is not needed. We would like to emphasize that variations in ECM content can also be observed when tissue sections are obtained from different levels along the muscle length since internal tendons may or may not be present [8]. In the “GlobalResults” file for this analysis, two outputs are reported: the ECM area (in µm^2^) and the percentage of the total section area accounted for the ECM area (Fig. 2A).

#### *Vascularization (*Fig. 2B*)*

The second feature of the panel is the assessment of the *Vascularization* of the skeletal muscle. In the original version of MuscleJ, the number of vessels was quantified and reported to their associated fibers [1]. Such staining corresponds to capillaries. We now distinguish between total *Vascularization*, including all types of vessels without morphological criteria, and *Capillaries* (detailed below in the section *Data Analysis by Fiber)*. This concerns all arteries or veins contained in the entire muscle section. This option measures the percentage of the total surface occupied by the vessels relative to the total section area of skeletal muscle. The number of vessels per mm^2^ is also provided in the result tables (Fig. 2B). Therefore, it is possible to perform an analysis of vascularization independently without using fiber morphology (which does not need to be labeled). As for the ECM, endothelial cell staining (for example, with CD31 antibody) is sufficient and is included in the algorithm to detect the muscle section; therefore, this channel can be used for the *Section Shape*.

#### *Specific Cells (*Fig. 2C*)*

Skeletal muscle tissue contains a variety of nonmyogenic cell types that are located between fibers and are not capillary or satellite cells, which are already tracked by MuscleJ. The third functionality is the characterization of these *Specific Cells* located anywhere in skeletal muscle. These may be, for example, resident stromal cells or infiltrating immune cells observed in pathological conditions or in injured tissues [9–11]. The name of the marker used to label-specific cells is entered manually as *Cell Marker* in the second dialog box (*Channel Information*) (Fig. 1F). This name will then be reported in the final table of results (Fig. 2C). This cell-specific marker can label an antigen as being located in the cytoplasm, membrane, or nucleus. A nuclear DNA label is also needed to ensure that the detected staining identifies a true cell and not artifacts such as cellular debris or a nonspecific signal. However, because nuclei of some cells may be imaged out of focus, the total number of specific cells, including those not counterstained with nuclear dye, is reported in the final “GlobalResults” file (*Nb-Specific Cells* and *Nb-Specific Cells with nuclei*) (Fig. 2C). In addition, MuscleJ2 provides information corresponding to the percentage of the area occupied by these cells (*%Specific Cell Area*), as well as the mean intensity of the signal in specific cells with nuclei (*Intensity Mean*) and their *Area Mean* (Fig. 2C).

Because each signal is different, MuscleJ2 provides the users with the raw data to allow them to set a personal threshold and filter their results based on their experience. In the final “SpecificDetails” file, the user can find the min and max Feret diameter, the coordinates (*x*, *y*) of the gravity center of the specific staining (only cells costained with DAPI), the nuclear center of gravity (*x*, *y*), and the intensity of the appropriate channel for each specific cell. As with all the other options, to allow viewing of the specific cells identified by MuscleJ2, their coordinates are saved in the ROI dedicated folder, and it is easy to return to any cell if needed. As an example, this new functionality was tested to detect F4/80-positive pan-macrophages (Fig. 2C) in a series of cross sections of regenerating muscle. Any validated antibody giving rise to a distinct signal in any cell in skeletal muscle can be used, offering a large panel of data analysis. In a set of images, it is possible to quantify several cellular markers, albeit one at a time, by running the batch of images for each specific marker. Since cells positive for multiple labels will share the same located nucleus, they could be quantified by mixing (using open-access software such as R) all the files “SpecificDetails” for each muscle section by the column *nucleus* *gravity center* (*x*, *y*).

All these novel functions are compatible with the other functions of the *Data Analysis by Fiber* panel.

### New functionalities reported for muscle fibers

**Fig. 3:**
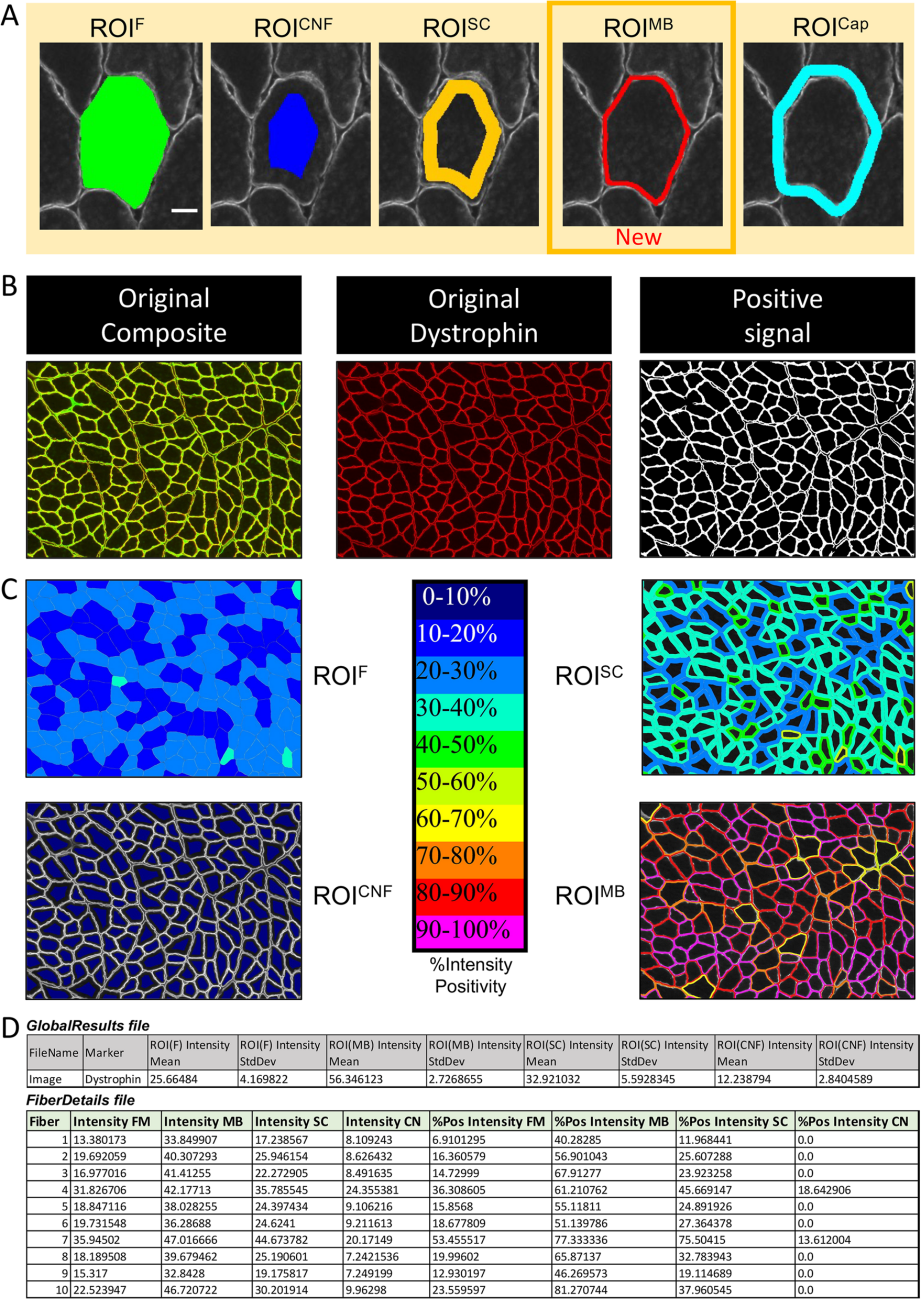
Measurement of Fiber Intensity by ROI. **A** Representation of the different ROIs in MuscleJ2. ROI^F^, ROI Fiber; ROI^CNF^, ROI Centronucleated Fiber; ROI^SC^, ROI Satellite Cell; ROI^Cap^, ROI Capillary; ROI^MB^, ROI Membrane. **B** Original image of skeletal muscle stained with dystrophin and corresponding cartographies representing the different ROIs obtained after MuscleJ2 analysis with the *Fiber Intensity* option. **C** For each fiber, the intensity of the staining and the percentage of positive pixels in each ROI are given. **D** Quantification of dystrophin staining in the different ROIs. The gray table presents the results obtained in the "GlobalResults" file, and the green table presents some of the results obtained in the "FiberDetails" file

In this section, all the results are given per fiber, based on the laminin staining (or any equivalent staining to identify myofibers). We have already described the different ROIs in the original version of MuscleJ [1], and they are conserved in this new version of the plugin. However, to be more precise, we have changed ROI^V^ (vessels) to ROI^Cap^ (capillaries), as explained previously. Moreover, we added a new ROI corresponding to the cellular membrane region of the fiber (ROI^MB^) (Fig. 3A). This new specific ROI^MB^ has been designed to quantify fluorescence staining in sarcolemmal or subsarcolemmal regions, such as the dystrophin-glycoproteins complex, where mutations in the genes encoding for its components can cause several muscular dystrophies.

We have also implemented new functionalities in this section.

#### Peri-myonuclei

Nuclei located inside myofibers are named “myonuclei” (Fig. 1). This novel functionality allows the quantification of the nuclei belonging exclusively to muscle fibers independently of the *Centro-Myonuclei* function. In healthy conditions, these nuclei exhibit a peripheral location. Since skeletal muscle is a highly adaptable tissue, their number may vary and needs to be quantified for each fiber. Myonuclei can be labeled in vivo using a transgenic mouse strain expressing histones coupled to GFP specifically in myofibers [12] or by using an antibody against the centrosomal protein PCM1 [13]. While PCM1 can also be expressed by proliferating myoblasts and macrophages in damaged muscle [14], MuscleJ2 can specifically detect myonuclei based on their location in the ROI^MB^ (Fig. S4).

To be identified as peripheral myonuclei by MuscleJ2, nuclei must be colabeled with the myonuclei marker and a fluorescent DNA stain such as DAPI. This is different from *Centro-Myonuclei* detection, which uses ROI^CNF^ and does not require colabeling because the central location may be sufficient for classification as myonuclei.

This analysis could be particularly useful to study myonuclei modifications in response to exercise training, and its controversial persistence during detraining (reviewed in Rhamati et al. [15]), which could vary according to fiber type, could be associated with changes in nuclei [16] or could be regulated by epigenetic modifications that could be investigated in situ with fluorescent labeling [17].

#### Capillaries

The option named *Capillaries* replaces the option *Vessels* of the original version of MuscleJ. This allows the user to analyze the capillaries associated with the fibers independently of the total vascularization of the muscle, which can now be performed using the *Vascularization* option, as described above. Consequently, the new ROI^Cap^ replaces the previous ROI^V^.

The “GlobalResults” file shows the number of fibers with capillaries and the total number of capillaries. The min and max Feret diameter, the gravity center coordinates (*x*, *y*), and the intensity of the appropriate channel for each capillary (Fig. 2B), as well as the parameter named *Sharing Factor* (SF), which represents the number of fibers around each capillary [17], are included in the “CapillaryDetails” file. In the “FibersDetails” file, the number of capillaries surrounding each fiber has been named *capillary contacts* to correspond to the commonly used terms [18, 19].

#### New fiber type IIX and changes in fiber typing

In the previous version of MuscleJ, fibers expressing type IIX myosin heavy chain (MyHC) were detected indirectly as corresponding to unstained fibers. In MuscleJ2, a channel can now be selected to directly identify this additional adult MyHC. This allows more accurate detection of type IIX fibers and hybrid myofibers expressing two or more isoforms [20]. This option is named *Type IIX* fibers (Fig. S5A). This allows, for example, investigation of hybrid myofiber transitions in disease or in response to exercise [20]. Specific labeling of fibers expressing MyHC IIX may be particularly useful for human muscle samples because the type IIB isoform is not expressed, and some antibodies may cross-react against other isoforms [21].

In addition, many changes were made in the fiber typing to improve this quantification (see “Methods”). The fiber-type analysis of the plugin has been optimized using a set of images from different users where type IIB or IIX fibers have, in most cases, a lower fluorescence intensity, probably due to lower reactivity of the IgM subclass of these primary antibodies [22]. We would like to point out that a fiber type could be variable along the same myofiber, as type IIA has been reported to be more abundant at the proximal extremity of the *tibialis anterior* in mice [3].

The thresholds are given in the “GlobalResults” file, as along with the associated fiber type defined by MuscleJ2 (Fig. S5B). However, if staining problems are encountered, it is possible not to use the automatic classification performed by MuscleJ2 and to go back to the “FiberDetails” files to reclassify the fibers manually based on a user-defined threshold.

#### *Fiber intensity by ROI (*Fig. 3*)*

A myriad of fluorescent labels can be investigated in muscle fibers as part of skeletal muscle research. The *Fiber Intensity by ROI* is a feature that allows quantification of any staining in muscle fibers (Fig. 1). Staining intensity is measured simultaneously in different areas of interest, since some markers may be heterogeneously expressed within the myofiber or at or below the cell membrane (sarcolemma). The results provide the intensity of the labeling and the %intensity positivity in the different ROIs (Fig. 3A–B). In the “GlobalResults” file, the average intensity of all segmented fibers is given for each ROI (*ROIx Intensity Mean*), as well as the associated standard deviation (*ROIx Intensity StdDev*).

For example, in regenerating or pathologic states, developmental isoforms could be re-expressed as embryonic and perinatal MyHC [23]. The quantification of the number of newly regenerated fibers re-expressing embryonic MyHC (MYH3 -positive fibers) can now be detected using this new function. Another example is the quantification of the percentage of dystrophin positivity in the different fiber ROIs, particularly in the ROI^MB^ (Fig. 3C). In the “GlobalResults” file, MuscleJ2 indicates the mean intensity of staining for all the fibers based on the staining/background ratio. However, the user can decide to use a different threshold based on the results by working directly on the “FiberDetails” file (Fig. 3D).

### Multiple analysis in the cartography section

**Fig. 4:**
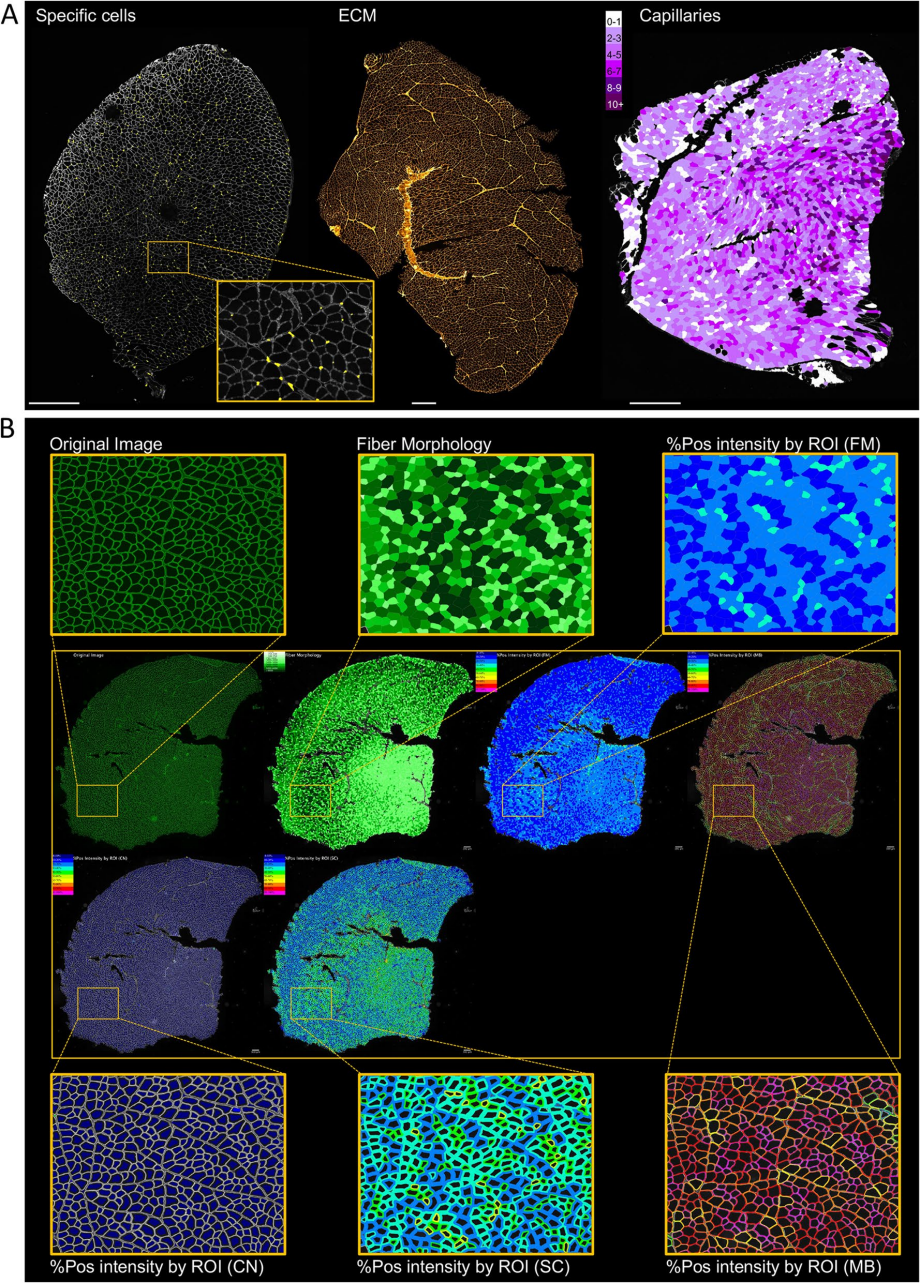
Novelty of the cartography section. **A** Representative images of the cartographies obtained for specific cells, ECM detection, and capillaries (*SB* = 600 µm). **B** Representation of the image obtained after selection of the multicartography option, in which different cartographies are assembled on the same image. In this example, the image was stained with dystrophin, and the results are represented in the different cartographies (*SB* = 300 µm)

All analyses carried out by MuscleJ2 can be visualized on cartographies in the *Data Cartographies* panel (Fig. 1). In addition to the five cartographies initially developed in MuscleJ to visualize the results of the analysis of *Fiber Morphology*, *Centro Myonuclei Fibers*, *Satellite Cells*, *Vessels*, and Fiber Type, we have added the cartographies of *peri-myonuclei*, *Fiber Intensity*, *Specific Cell Localization*, and in situ* ECM* *Signal *(Fig. 4A). MuscleJ2 also offers the possibility of adding a legend and a scale bar at different positions of the image, determined by the user. In addition, it is now possible for the user to select the channel on which the cartography will be drawn (in the second dialog box: *Image used for cartographies*). Furthermore, for the option *Fiber Intensity by ROI*, different cartographies were added to represent the MuscleJ2 results in the different ROIs. Another new option in this panel, named *Multi-Cartography Montage*, allows users to obtain a photo montage of all selected options in the *Data cartography* panel (Fig. 4B).

### Generation of metadata files

After each run, MuscleJ2 generates a text file containing a summary of the options and selected analyses (Fig. S6). This file allows the user to easily retrieve the metadata associated with the performed analysis and is in the result folder along with other files such as “GlobalResults” and “FiberDetails.” In the latter, the user can access the details of the requested *Analysis by Fiber*. The user can therefore use the "ROI" file to review the identified fibers of the section and possibly manually delete some major aberrant fiber detections. However, we do not recommend adding new fibers manually, as this will add an additional source of variability since the mode of quantification will be different from that performed automatically. The “GlobalResults” file averages all the fibers in the section. Compared to the original version of MuscleJ, it is no longer generated at the end of the run but is updated after each executed image to obtain the global results step by step, without losing data if the plugin unexpectedly stops before the end of the process.

## Discussion

The development of MuscleJ2, running on ImageJ/Fiji, now significantly extends the functionality of the original macro to cover a wider range of possible quantification scenarios performed on fluorescence-labeled images of skeletal muscle sections.

While numerous other software programs have been developed since the publication of the original MuscleJ, most of them are complementary and focus on specific parameters (Sup. Table 1). One of the major advantages of MuscleJ2 over other comparable software is its capacity to handle a combination of different types of analysis. All the described functions can be used together to provide new information.

With the parallel development of new acquisition systems, which offer the possibility of working with more than four fluorescent markers, MuscleJ2 enables multioutput skeletal muscle analysis. For example, it is possible to perform multiple labeling with the detection of laminin and nuclei associated with three types of myosin heavy chains, requiring 5 detection channels (Fig. S4) or more complex multicolor staining with codetection of laminin, vessels, and two types of myosin heavy chains using one of the protocols described by Bailly et al. [24].

The developed plugin is easy to use, and the troubleshooting annex of the User Guide will be completed based on feedback received from users to list the problems users encounter and their solutions. We encourage users to keep up to date with the changes that will be made in future versions of MuscleJ2, which will be updated and made available on GitHub.

We will continue to implement new features in the plugin, which will be updated online and detailed on GitHub. All new features to be developed will be available on GitHub, which will be updated regularly.

## Methods

### Animals and tissue preparation

To validate and illustrate the new features of MuscleJ2, skeletal muscle sections were obtained from mice and rats. Animals underwent experimental procedures approved by local ethics committees for other projects in which tissue sampling had already been planned. Rats were euthanized by decapitation following isoflurane anesthesia. For both models, various muscle anatomic localizations were harvested, including the hind limb (*tibialis anterior* and *gastrocnemius*) as well as the diaphragm and snap-frozen in liquid nitrogen-cooled isopentane. Samples were then stored at − 80 °C before cryosectioning.

### Immunofluorescence staining protocols

Immunofluorescence staining was performed on frozen thin sections of skeletal muscle (7 to 12 μm). Briefly, sections were rehydrated with 1X PBS and fixed for 10 min (mouse samples) and 20 min (rat) in 4% (w/v) paraformaldehyde (PFA) in 1X PBS. After washing in 1X PBS, the cells were permeabilized with 0.1% or 0.5% (v/v) 1X PBS-Triton × 100 and then blocked either in 1X PBS –5% horse serum (mouse samples) or 10% BSA (F4/80 staining in mouse samples) or else in Emerald: Antibody Diluent (no. 936B-08, Sigma; rat samples). Primary antibodies were incubated overnight (ON) at 4 °C. Hoechst H33342, and WGA and secondary antibodies, were incubated for 45 min at room temperature. In rat samples, nuclei were stained by DAPI contained in the mounting medium.

The specific protocols, products, antibodies, and markers used are described in Sup. Table 2. Most of the images were acquired at 10 × and 20 × magnification of the whole section using the Axio Scan. Z1 (Zeiss, Germany), in several imaging platforms, includes one with 7 fluorescence channels by LED light source (385 nm/430 nm/475 nm/555 nm/590 nm/630 nm/735 nm). Some acquisitions were performed on the NanoZoomer S60 equipped with 5 fluorescence channels (Hamamatsu, Japan) or on the epifluorescence microscope DM6000 (Leica, Germany) using a monochrome camera.

### Programming environment

#### MuscleJ2 as a plugin in Fiji/ImageJ

As Java-based public domain software implemented as a plugin for ImageJ (NIH, Bethesda, MD, USA, https://imagej.nih.gov/ij/) or Fiji [25], MuscleJ2 benefits from the facilities offered by Fiji/ImageJ for image input/output and preprocessing.

The Java source codes were developed in the *Eclipse IDE for Java Developers* free environment (Version 4.23.0, www.eclipse.org) with ImageJ internal libraries.

Thanks to the Java language, memory management has been optimized, and MuscleJ2 permits either a larger batch or image size than the previous macro MuscleJ. Moreover, several features have been optimized to increase the speed of *Analysis by Fiber*, e.g., for morphological analysis, the time mean by section with MuscleJ2 is decreased by 2 and that for result cartographies by 10.

#### Hardware and software requisites

The plugin has been tested on different operating systems (OS) such as Windows7, 8, and 10/MacOS Monterey up to 12.4/Ubuntu 20.04 with the following minimum computer requirements:

- RAM: 8 GB minimum, 16 GB highly suggested
- System type: 64-bit operating system

The Fiji/ImageJ environment is required with a maximum memory setting fixed to 75% of the computer’s total memory, and the *Bio-Formats* plugin (https://docs.openmicroscopy.org/) must be present in the *Plugins* menu.

The plugin has been tested on the following software versions:

- Fiji/ImageJ version: from 1.51e to 1.53t
- Java version (64 bits): From Java 1.8.0–66 to Java 1.8.0–172
- Used plugins: Bio-Formats plugins (up to release 6.6)

For more information about MuscleJ2 plugin installation and preliminary requests before starting MuscleJ2, please refer to the *User’s Guide Chap. I* “MuscleJ2 in the Fiji/ImageJ environment.”

### New implementations and improvements

The main entry point of MuscleJ2 is a graphical user interface organized into five panels: *Sample Data, Data Acquisition, Data Analysis by Section, Data Analysis by Fiber*, and *Data Cartographies* involving the implementation or improvement of several functions related to these different panels.

#### User interface panel 1: sample data

##### Option Pathophysiology:

The plugin measures the mean of all the CSA fibers of the section. From this average, only fibers with a circularity between 0.45 and 1 and a fiber area between 100 µm^2^ and area mean + 3 × StdDev for the *Healthy* option and 50 µm^2^ and area mean + 4 × StdDev for the *Damaged* option are analyzed.

#### User interface panel 2: analysis by section

##### Option ECM Area Detection:

To detect the area representative of ECM, a threshold based on *Moments* method was performed, followed by a low erosion filter.

##### Option Vascularization:

After a series of pretreatments and an intensity histogram analysis to subtract the background and to detect the real intensity on the appropriate channel, the vessel borders are delimited, and the total surface covered by vessels is calculated.

The *Vascularization surface (%)* mentioned in the “GlobalResults” table corresponds to the ratio between the total surface covered by vessels and the total surface of the section.

##### Option Specific Cells:

The algorithm first applies a series of pretreatments on the indicated channel to quantify both circular and irregularly shaped cells on the whole section. Then, the nuclei are localized on the appropriate channel, and MuscleJ2 checks if they overlap with the specific cells previously segmented. At this step, there are two sets of specific cells, with or without nuclei gravity center coordinates, as mentioned in the “SpecificDetails” file.

#### User interface panel 3: analysis by fiber

##### ROI^MB^ definition:

The ROI^MB^ is defined as the space inside the fiber ROI corresponding to one-twentieth of the minimal (Min) Feret diameter of the ROI^F^.

##### Option capillaries:

MuscleJ2 recognizes capillaries with specific morphological criteria, such as maximum surface (100 µm^2^) and minimum circularity (0.45).

##### Option fiber Intensity by ROI:

To track the real positive signal inside the ROIs, after background subtraction, an automatic threshold based on intensity histogram analysis was applied to attribute each fiber a percentage of positive intensity defined by the number of positive intensity pixels divided by the surface of the ROIs (%intensity positivity) (Fig. 3B).

##### Option fiber typing:

With this option, for all fibers detected by morphological analysis, the intensity mean (mean_IF_) and its standard deviation were calculated by channel corresponding to a type of fiber (I, IIA, IIB, or IIX).

Based on intensity histogram analysis, different thresholds of positivity, depending on fiber types, have been defined as Mean_IF_ + StdDev for type I and IIA fibers and equal to Mean _IF_ for type IIB and IIX fibers.

#### User interface panel 4: data cartographies

##### Option: legend

The sizes of the legend (width and height) are proportional to the original image size. For the type legend, only the hybrid types present in the section are mentioned.

##### Option scale bar:

With this option, four locations to position the scale bar are possible: lower right, lower left, upper right, and upper left. By default, no scale bar is shown. The length of the scale bar is fixed to 300 µm for a whole section and to 100 µm for an image crop.

##### Option Multi Cartography Montage:

For this option, thanks to the ImageJ function called “Make a montage…”; an automatic montage of the asked cartographies is created with the following parameters: if the number of cartographies is higher than 3, a new line is created to perform readable montage with a reasonable size. A title is written on each cartography corresponding to the analysis performed.

For more information on the features and options of the MuscleJ2 plugin, please refer to the *User Guide Chap. II* “How to launch MuscleJ.”

### Output refinement

#### Metadata and user choices by batch

A text log file by batch is created with the following nomenclature: *YYYYMMDD_HHMMSS_imagefoldername_*BATCH_LOG.txt.

It contains the information associated with the performed analysis as the metadata selected by the user in the principal dialog box but also the performed analysis, channel attribution, and general information linked to the batch run.

#### Data analysis nomenclature

A text global result file by batch run is created with the nomenclature "*ImageFolderName*_GlobalResults_*Listofanalysisperformed*.txt", where “*ImageFolderName*” corresponds to the name of the image folder selected at the beginning of the batch run and “*Listofanalysisperformed*” corresponds to the abbreviations added at the end of the global result file name. This allows the user to associate a global result table with the analysis pipeline performed by batch run.

#### Output files

Each distinct cell type has a proper file such as “SatCellDetails” for satellite cells, “SpecificDetails,” “fluorescent marker name” for each labeling of specific cells, or “CapillaryDetails” replacing the “VesselDetails” file of the previous version. All these files have been provided for each image analyzed per batch run (for more information, see *User Guide Chap. III* “Description of result files by batch”).

All fiber ROIs are automatically saved in the ROI folder with the extension “_xxROI.zip,” including the ROI corresponding to section shape but also the ROIs for specific cells as well as for satellite cells.

### Supplementary Information

**Additional file 1:** **Fig. S1.** Comparison of the *Homogeneous* and *Heterogeneous* options. Comparison of the results and cartographies obtained by MuscleJ2 after fiber morphology analysis on laminin-stained *tibialis anterior* sections after partial injury, with the *Healthy* option (left) and the *Damaged* option (right). Histograms at the bottom show the frequency of fibers with different cross-sectional areas. The results show that the number of included fibers is higher with the *Damaged* option than with the *Healthy* option, where numerous fibers are excluded from the analysis (black fibers). **Fig. S2.** Analysis of the diaphragm with MuscleJ2. **A** Cartography of the extracellular matrix (ECM) staining representing the diaphragm of the mouse rolled up on itself at the time of cutting. **B** Comparison of the results obtained by MuscleJ2 after ECM analysis on laminin-stained diaphragm sections with the *Diaphragm* option and the *Limb* option. The results show that the total surface area is smaller with the *Diaphragm* section due to the removal of the fiberless parts (*), which is not the case with the *Limb* option. **Fig. S3.** Comparison of the Data Acquisition panel between the macro and the plugin MuscleJ2. Screenshots of the dialog boxes in the initial macro (**A**) and the plugin (**B**) showing changes in the Data acquisition section. **Fig. S4.** Peri-myonuclei detection with MuscleJ2. A Skeletal muscle section from injured mouse (21 days post-injury) stained with DAPI, PCM1 and laminin antibodies (SB=500 µm) and representative cartography of perimyonuclei quantification results obtained with MuscleJ2. Only double PCM1/DAPI-positive cells in the ROI^MB^ are quantified and represented in the cartography. **B** The gray table shows the results obtained from the "GlobalResults" file, and **C** The green table shows a subset of the results obtained from the "PeriMyoNucleiDetails" file. **Fig. S5.** Fiber typing with MuscleJ2. **A** Skeletal muscle section stained with DAPI, myosin heavy chain IIX, IIA, I and laminin antibodies (SB=600 µm). **B** Representative cartographies (original image, fiber morphology and fiber typing) of the results obtained with MuscleJ2 (SB=300 µm). **C** Global file results and fiber detail results. All areas are given in µm^2^. **Fig. S6.** Global information associated with a MuscleJ2 batch run. Screenshots of the BATCH_LOG text file corresponding to the meta data and the analysis performed during the batch run as well as channel attributions to keep a printout of options and global information. **Sup. Table 1.** Main features of tools and software available to perform a fully or semiautomatic analysis on skeletal muscle immunofluorescence images. List of abbreviations used: AI: artificial intelligence; DPI: days post injury; EBD: Evans blue dye; IF: immunofluorescence; IHC: immunohistochemistry; MYH: myosin heavy chain (gene); NCAM: neural cell adhesion molecule; PFA: paraformaldehyde; ref: reference. **Sup. Table 2.** List of antibodies and specific protocols used.

## Data Availability

A portion of the data that support the findings of this study are available on the shared GitHub space, but restrictions apply to the availability of some images; therefore, they have been anonymized. All the data corresponding to the described analysis can be downloaded from the GitHub site, but they cannot be used without request to the corresponding authors.

## Abbreviations

BSA: Bovine serum albumin
Cap: Capillary
CNF: Centronucleated fiber
CSA: Cross-sectional area
DAPI: 4′,6-Diamidino-2-phenylindole
DNA: Deoxyribonucleic acid
ECM: Extracellular matrix
F: Fiber
FAP: Fibro-adipogenic progenitor
Ig: Immunoglobulin
MB: Membrane
Min/max: Minimal/maximal
MYH: Myosin heavy chain (gene)
MyHC: Myosin heavy chain (protein)
ON: Overnight
PBS: Phosphate-buffered saline
PCM1: Pericentriolar material 1
PFA: Paraformaldehyde
ROI: Region of interest
SB: Scale bar
SC: Satellite cell
StdDev: Standard deviation
V: Vessel
v/v: Volume per volume
w/v: Weight per volume
WGA: Wheat germ agglutinin
