# Supplementary material for: MuscleJ2: a rebuilding of MuscleJ with new features for high-content analysis of skeletal muscle immunofluorescence slides
Source: Skelet Muscle. 2023 Aug 23;13:14. doi: 10.1186/s13395-023-00323-1 (PMC10463807; doi:10.1186/s13395-023-00323-1)
Supplement: Supplementary file 1 — Additional file 1: Fig. S1. Comparison of the Homogeneous and Heterogeneous options. Comparison of the results and cartographies obtained by MuscleJ2 after fiber morphology analysis on laminin-stained tibialis anterior sections after partial injury, with the Healthy option (left) and the Damaged option (right). Histograms at the bottom show the frequency of fibers with different cross-sectional areas. The results show that the number of included fibers is higher with the Damaged option than with the Healthy option, where numerous fibers are excluded from the analysis (black fibers). Fig. S2. Analysis of the diaphragm with MuscleJ2. A Cartography of the extracellular matrix (ECM) staining representing the diaphragm of the mouse rolled up on itself at the time of cutting. B Comparison of the results obtained by MuscleJ2 after ECM analysis on laminin-stained diaphragm sections with the Diaphragm option and the Limb option. The results show that the total surface area is smaller with the Diaphragm section due to the removal of the fiberless parts (*), which is not the case with the Limb option. Fig. S3. Comparison of the Data Acquisition panel between the macro and the plugin MuscleJ2. Screenshots of the dialog boxes in the initial macro (A) and the plugin (B) showing changes in the Data acquisition section. Fig. S4. Peri-myonuclei detection with MuscleJ2. A Skeletal muscle section from injured mouse (21 days post-injury) stained with DAPI, PCM1 and laminin antibodies (SB=500 µm) and representative cartography of perimyonuclei quantification results obtained with MuscleJ2. Only double PCM1/DAPI-positive cells in the ROIMB are quantified and represented in the cartography. B The gray table shows the results obtained from the "GlobalResults" file, and C The green table shows a subset of the results obtained from the "PeriMyoNucleiDetails" file. Fig. S5. Fiber typing with MuscleJ2. A Skeletal muscle section stained with DAPI, myosin heavy chain IIX, IIA, I and laminin anti [file 13395_2023_323_MOESM1_ESM.pdf]

Supplementary file

MuscleJ2: a rebuilding of MuscleJ with new features for high content analysis of skeletal muscle immunofluorescence slides

Anne Danckaert[1]*, Aurélie Trignol[2], Guillaume Le Loher[1,3], Sébastien Loubens[4,5], Bart Staels[4], Hélène Duez[4], Spencer L. Shorte[1], Alicia Mayeuf-Louchart[4]*

1. Institut Pasteur, Université Paris Cité, UTechS Photonic BioImaging/C2RT, F-75015 Paris, France
2. French Armed Forces Biomedical Research Institute (IRBA), France; Université Paris Cité, VIFASOM (UPR 7330 Vigilance Fatigue, Sommeil et Santé Publique), France.
3. Present Address: École Centrale d'Electronique (ECE), Paris, France.
4. Univ. Lille, Inserm, CHU Lille, Institut Pasteur de Lille, U1011- EGID, F-59000 Lille, France
5. CHU Lille, Service Neuropédiatrie, 59000 Lille, France

*Corresponding authors: anne.danckaert@pasteur.fr; alicia.mayeuf-louchart@inserm.fr

Figure Legends and Tables

Fig. S1.Comparison of the *Homogeneous* and *Heterogeneous* options. Comparison of the results and cartographies obtained by MuscleJ2 after fiber morphology analysis on laminin-stained *Tibialis anterior* sections after partial injury, with the *Healthy* option (left) and the *Damaged* option (right). Histograms at the bottom show the frequency of fibers with different cross-sectional areas. The results show that the number of included fibers is higher with the Damaged option than with the *Healthy* option where a number of fibers are excluded from the analysis (black fibers).

Fig. S2. Analysis of the diaphragm with MuscleJ2. (A) Cartography of the extracellular matrix (ECM) staining representing the diaphragm of the mouse rolled up on itself at the time of cutting. (B) Comparison of the results obtained by MuscleJ2 after ECM analysis on laminin-stained diaphragm sections, with the *Diaphragm* option and the *Limb* option. The results show that the total surface area is smaller with the *Diaphragm* section due to the removal of the fiberless parts (*), which is not the case with the *Limb* option.

Fig. S3. Comparison of the Data Acquisition panel between the macro and the plugin MuscleJ2. Screenshots of the dialog boxes in the initial macro (A) and the plugin (B) showing changes in the Data acquisition section.

Fig. S4. Perimyonuclei detection with MuscleJ2. (A) Skeletal muscle section from injured mouse (21days post-injury) stained with Dapi, Pcm1 and laminin antibodies (SB=500µm) and representative cartography of perimyonuclei quantification results obtained with MuscleJ2. Only double Pcm1/Dapi-positive cells in the ROI^{MB} are quantified and represented in the

cartography. (B) The grey table shows the results obtained from the global results file and (C) the green table a subset of the results obtained from the *PeriMyoNucleiDetails* file.

Fig. S5. Fiber typing with MuscleJ2. (A) Skeletal muscle section stained with Dapi, Myosin Heavy chain IIX, IIA, I and laminin antibodies (SB=600µm). (B) Representative cartographies (original image, Fiber morphology and Fiber Typing) of the results obtained with MuscleJ2 (SB=300µm). (C) Global file results and fiber detail results. All areas are given in $µm^2$.

Fig. S6. Global information associated with a MuscleJ2 batch run. Screenshots of the BATCH_LOG text file corresponding to the meta data and the analysis performed during the batch run as well as channel attributions, to keep a printout of options and global information.

Sup Table 1: Main features of tools and software available to perform a fully or semi-automatic analysis on skeletal muscle immunofluorescence images.

List of abbreviations used: AI: artificial intelligence; DPI: days post injury; EBD: Evans blue dye; IF: immunofluorescence; IHC: immunohistochemistry; MYH: myosin heavy chain (gene); NCAM: neural cell adhesion molecule; PFA: paraformaldehyde; ref: reference.

Sup Table 2: List of antibodies and specific protocols used

FigS1.

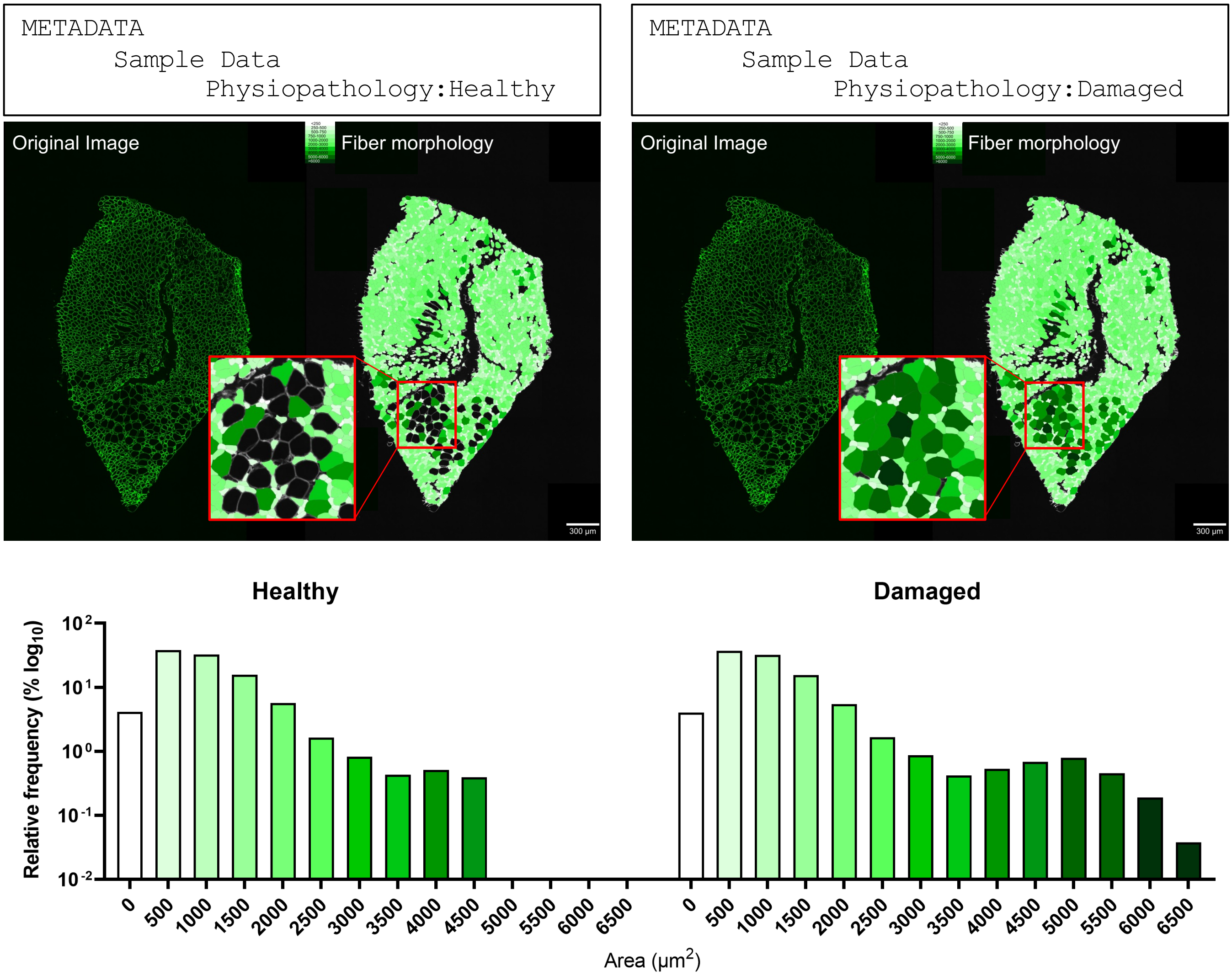
METADATA
Sample Data
Physiopathology:Healthy
METADATA
Sample Data
Physiopathology:Damaged
Original Image
Fiber morphology
300 µm
Original Image
Fiber morphology
300 µm
Healthy
Damaged
Relative frequency (% log10)
10^2
10^1
10^0
10^-1
10^-2
0 500 1000 1500 2000 2500 3000 3500 4000 4500 5000 5500 6000 6500
0 500 1000 1500 2000 2500 3000 3500 4000 4500 5000 5500 6000 6500
Area (µm2)

FigS2.

A

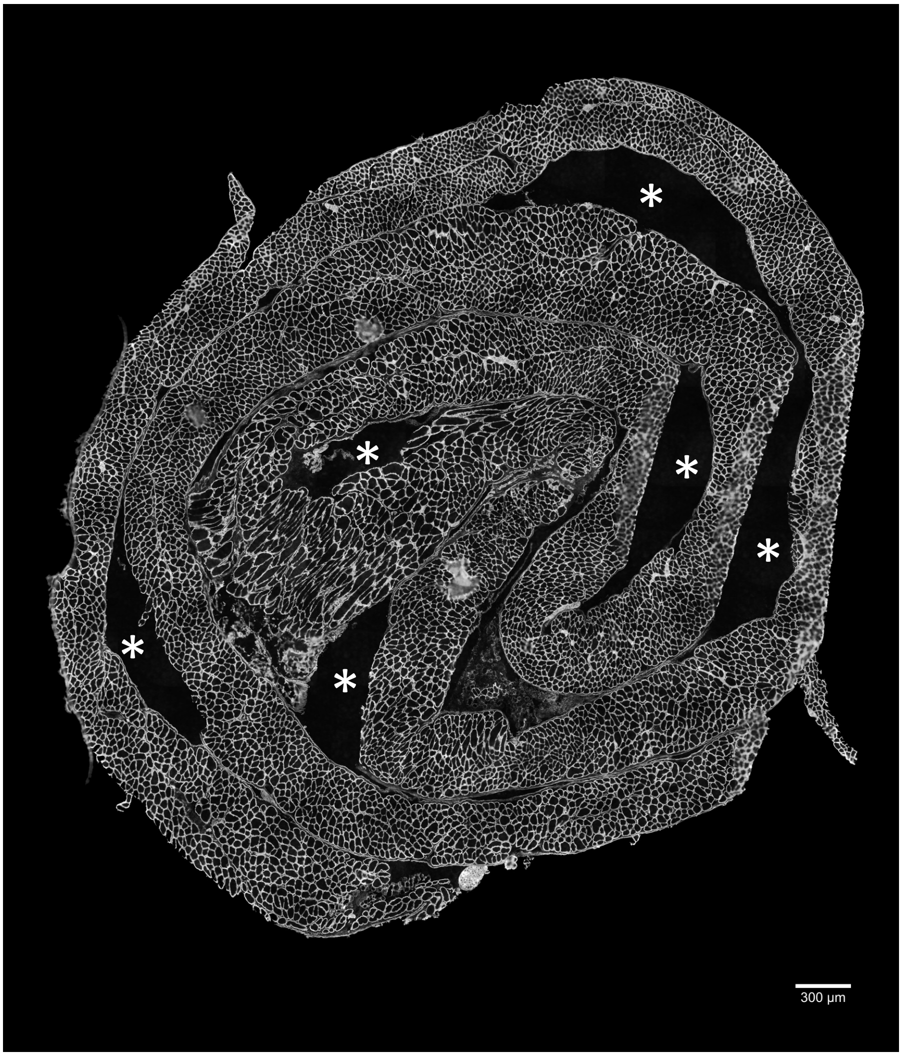

B

	Option Diaphragm	Option Limb
**Whole Section Area**	7785545.0	1.5096652E7
**ECM Area Tot**	1684250.4	1684294.8
**% ECMArea**	21.633043	11.156744

FigS3.

A

Macro Muscle J 1.0.1 (2018)

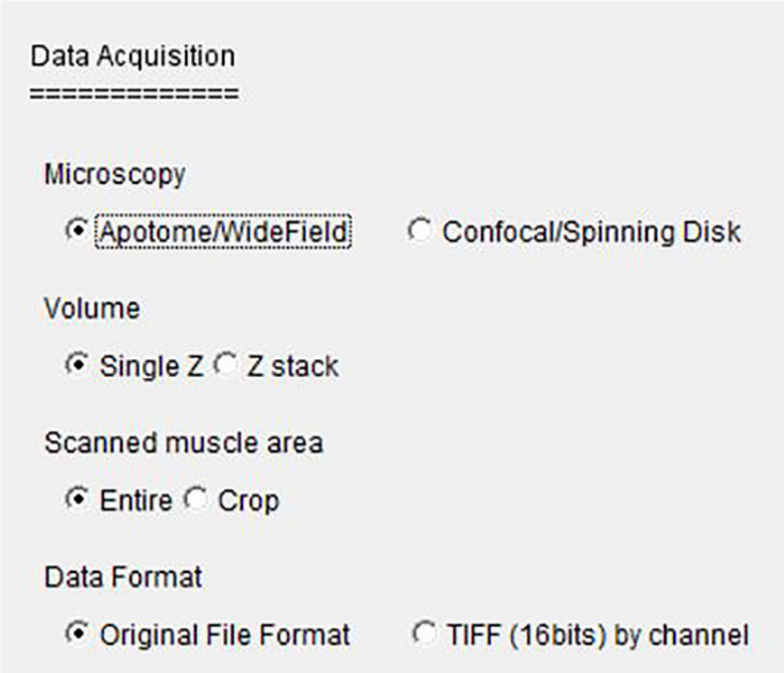

B

Plugin Muscle J 2.0 (2022)

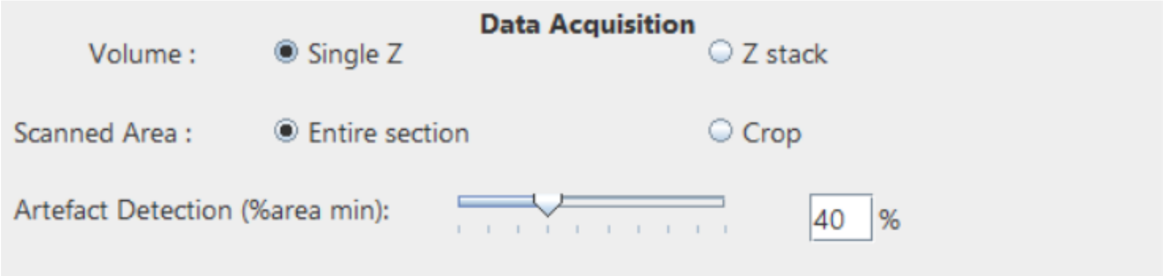

FigS4.

A

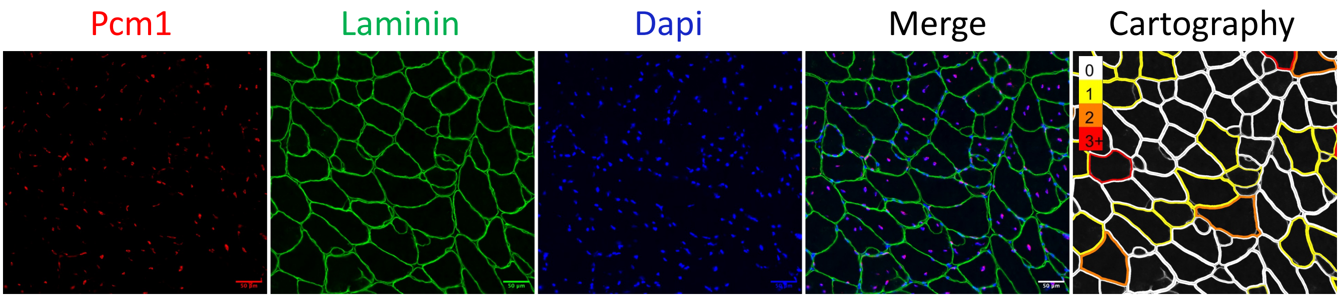

B

FileName	Whole Section Area	Nb Segmented Fiber	Nb Segmented Fiber	Nb Real PeriMyoNuclei
Image 1	7351347.5	3314	3314	9053

C

Perimynonuclei details	Area	Max Feret	Min Feret	Nuclei GC X	Nuclei GC Y	Intensity
1	47.32	9.825541	7.8	2253	537	3201.2812
2	71.4025	11.830178	8.953805	2367	550	4157.487
3	32.638126	9.475297	5.285623	2304	567	3148.1326
4	15.21	8.604832	3.71222	2297	574	2688.9307
5	26.82875	7.914267	4.665732	2115	575	3261.9211

FigS5.

A

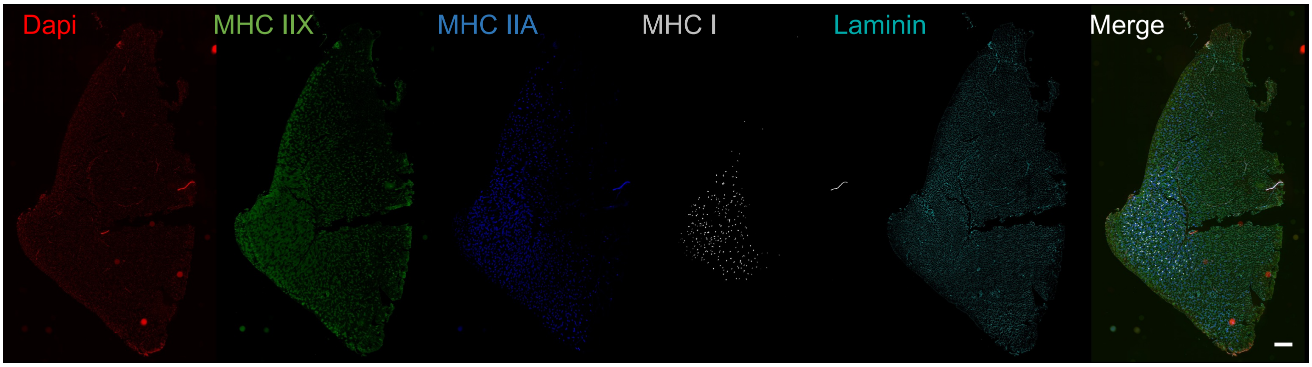

B

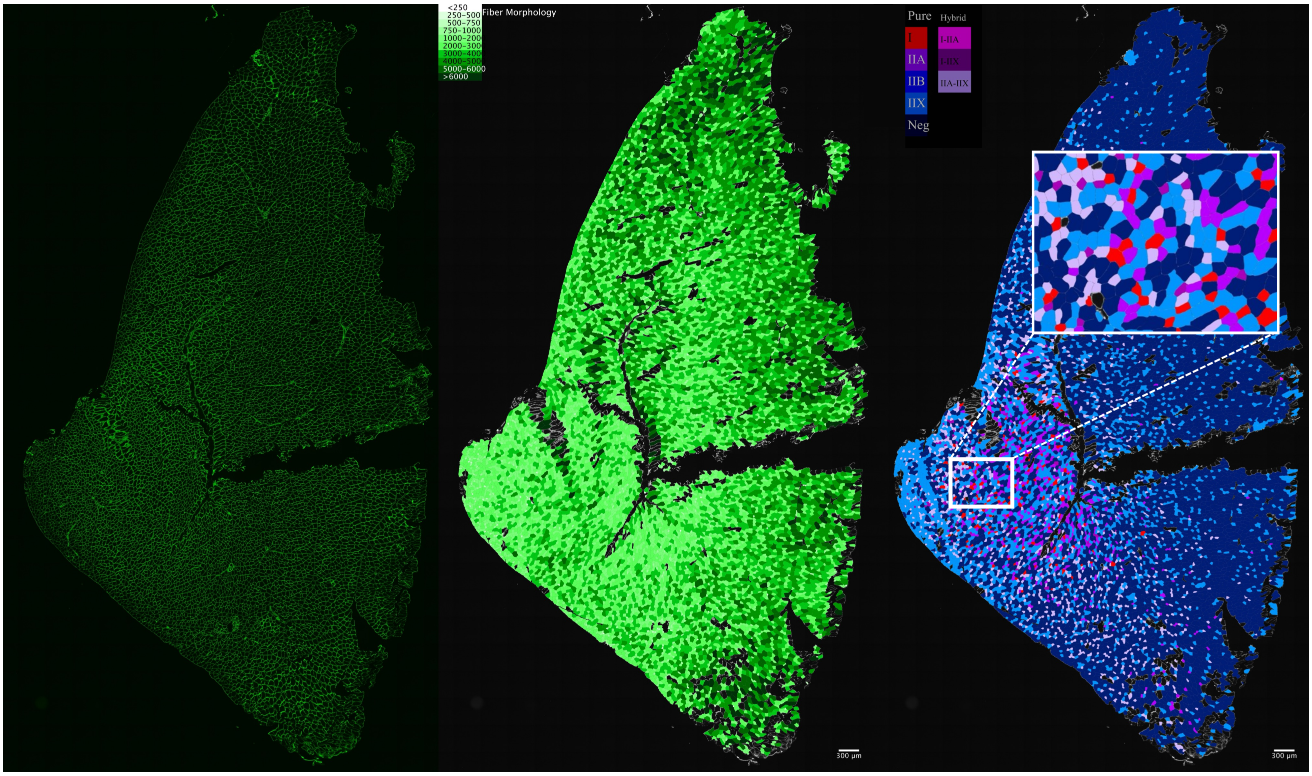

C

GlobalResults file

FileName	Whole Section Area	Nb Segmented Fiber	Nb Type I	Thres. I	Nb Type IIA	Thres. IIA	Nb Type IIX	Thres. IIX	Nb Type Neg	Nb Type I-IIA	Nb Type I-IIX	Nb Type IIA-IIX
Image	3.6394256E7	11501	161	3.1257656	567	9.799161	2848	1.3097754	6553	5	90	1303

FiberDetails file

Fiber details	Area	Intensity Type I	Intensity Type IIA	Intensity Type IIX	Fiber Type
3286	1385.0057	1.1461766	16.572737	1.5782285	IIA-IIX
3287	1162.2657	1.0533646	9.317839	2.2394698	IIX
3288	1499.1063	0.840229	15.722873	2.899948	IIA-IIX
3289	1632.9064	0.75740564	1.4910415	2.4351408	IIX
3290	1040.9486	18.883455	4.2055464	1.8933858	I-IIX
3291	1340.5358	1.0152771	19.135895	2.5192783	IIA-IIX
3292	1786.2108	0.8130596	8.119895	2.3356628	IIX
3293	2545.321	0.8429119	2.162912	2.2293487	IIX
3294	3860.5012	0.50881624	1.1416663	0.669277	Neg
3295	1527.5828	0.5188968	2.1446629	2.5491574	IIX
3296	1876.126	0.7485186	1.5382056	1.3699969	IIX
3297	2460.282	0.9463295	2.7606628	3.2271285	IIX

FigS6.

Images_BATCH_LOG.txt

```
METADATA
        Sample Data
                Physiopathology:Healthy
                Muscle Type:Limb
        Acquisition Data
                Volume: Single Z
                Scanned Area: Entire Section
                Artefact Detection limit: 40
PERFORMED ANALYSIS
        2 Analysis by Fiber
                Fiber Morphology
                Centro Nuclei Fibers
CHANNEL ATTRIBUTION
CHANNEL #2 for Fiber shape detection
CHANNEL #1 for Nuclei detection
GENERAL INFORMATION
        Image Folder Path: /Users/aml/Desktop/Images/
        Result Folder Path: /Users/aml/Desktop/Results/
Nb Sections analysed: 2
Nb Sections rejected: 0
```

Sup Table 1.

						Software features												
**Sofware (ref)**	**Year**	**ImageJ/FIJI**	**Sample preparation**	**Staining protocol**	**Human biopsies**	**Fiber morphology**	**Myonuclei**	**Central nuclei**	**Satellite cells**	**Capillaries**	**Fiber type**	**Myofiber staining**	**Sarcolemmal staining**	**ECM**	**Specific cells**	**Cartography**	**Conditions tested**	**Comments**
MyoScan (1)	2013		Frozen	IF/IHC		X		X									Healthy and mdx mice samples	Using web-based image analysis system S.CORE
Skeletal Muscle Algorithm (2)	2013		Frozen	IF staining		X					X	NCAM+					Mice (healthy and injured), rat, swine and monkey samples	Using CyteSeer
Algorithm by Beekman et al. (3)	2014		Frozen	IF staining	X	X							Dystrophin+, Spectrin+				Healthy human samples and patients with a myopathy	Using Definiens, providing dystrophin and sarcolemmal intensities
SMASH (4)	2014		Frozen	IF staining		X		X		X	X						Healthy and mdx mice	Using Matlab, providing capillaries density
MyoVision (5)	2018		Frozen	IF staining		X	Dapi staining				X						Mice muscle samples	
By Sardone et al. (6)	2018		Frozen	IF staining	X	X							Dystrophin+, Spectrin+, Laminin+				Healthy human samples and patients with a myopathy	Using Definiens, providing dystrophin and sarcolemmal intensities
MuscleJ (7)	2018	X	Frozen	IF staining		X	Dapi staining	X	X	X	X					X	Healthy, notexin-injected (21DPI) and mdx mice	
MuscleAnalyzer (8)	2018		Frozen	IF staining		X		X									Healthy and mdx mice samples	Using CellProfiler
Open-CSAM (9)	2019	X	Frozen	IF staining		X											Healthy, cardiotoxin-injured (8, 14 and 28DPI), mdx and old mice	
MuscleMap (10)	2019		Frozen	IF staining	X								Dystrophin+				Healthy human samples and patients with a myopathy	By Flagship Biosciences, providing also a positive-fiber classication and quantify the number of myofibers
Macro by Reyes-Fernandez et al.(11)	2019	X	Frozen	IF staining	X	X					X					X	Healthy human samples (young, old, obese) or with a myopathy	
QuantiMus (12)	2019		Frozen	IF staining	X	X		X			X	NCAM+, MYH3+, EBD+					Healthy and mdx mice; patients with a suspected myopathy	Using Flika (Python), AI algorithm to improve efficiency and speed
SLCV (13)	2019		Frozen	IF staining		X											Healthy mice samples	AI algorithm for various immunostaining qualities
Muscle2View (14)	2019		Frozen	IF staining	X	X	Dapi staining	X		X	X						Healthy human samples	Using CellProfiler, providing also capillarization indices
Myosoft (15)	2020	X	Frozen	IF staining		X					X					X	Healthy and mdx mice samples	Could be used with suboptimal stainins
By Scaglioni et al. (16)	2020		Frozen	IF staining	X	X						MYH3+, MYH8+	Dystrophin+, αSarcoglycan+, βDystroglycan+			X	Healthy human samples and patients with a myopathy	Using Definiens, providing also a positive-fiber classication and a % of sarcolemmal circumference coverage
MyoSight (17)	2020	X	Frozen	IF staining		X	Dapi staining	X			X						Healthy and mdx mice samples	Single software with multiple analyses, using 5 channels
MuscleJ modified by Bonilla et al.(18)	2020	X	Frozen	IF staining	X	X					X						Human samples (older aldults to study muscle aging)	
MyoSAT (19)	2020	X	Frozen	IF staining		X										X	Healthy and after nerve-injured mice samples; healthy dogs	For weak or non-uniform staining
MuscleJ modified by Bindellini et al.(20)	2021	X	Frozen	IF staining		X					X						Healthy mice samples	
LabelsToRois (21)	2021	X	Frozen (+/- PFA before)	IF staining		X										X	Mdx and injury models (cardiotoxin or glycerol at 21DPI) mice	Coupled to Cellpose (AI algorithm) for suboptimal stainings
MyoView (22)	2021		Frozen	IF staining		X	Dapi staining		X		X						Mdx and healthy mice (exercise-induced injury protocol)	Using Matlab
By Vetter et al. (23)	2021		Frozen	IF staining	X	X							Dystrophin+, Spectrin+, Laminin+			X	Healthy human samples and patients with a myopathy	Using NIS-Elements (Nikon), providing also a positive-fiber classication
MyoProfiler/SiriusProfiler (24)	2022		Frozen	IF/Sirius/Masson	X	X	Dapi staining	X	In theory					X	F4/80+		Healthy and mdx mice samples	Using CellProfiler
MyoVision 2.0 (25)	2022		Frozen	IF staining		X	Dapi staining +/- second staining (co-localization)				X						Healthy rat (electrical stimulation and atrophy protocols)	Using U-net (segmentation) and neural networks (fiber detection and fiber-type classification), providing myonuclear domain size

Sup Table 2.

Specific labelling	Primary antibody	Secondary antibody and others (45min RT)	Muscle sample
**ECM**			
Oligosaccharide residues	/	Wheat Germ Agglutinin Alexa Fluor 555 (#W32464, Invitrogen) 1:1000	Rat samples
**Vascularization/Capillaries**			
Endothelial cells	Mouse IgG1 monoclonal Anti CD31 (#ab64543, Abcam) 1:200 ON 4°C	Donkey anti Mouse IgG (H+L) Alexa Fluor 594 (#A21203, Thermo Fisher Scientific) 1:500	Rat samples
Laminin	Rabbit IgG polyclonal Anti Laminin (#ab11575, Abcam) 1:200 ON 4°C	Goat anti Rabbit IgG (H+L) Alexa Fluor 488 (#A21206, Thermo Fisher Scientific) 1:500	
**Specific cells**			
Macrophages	Rat IgG2b monoclonal Anti F4/80 (#MCA497G, Bio-Rad) 1:100 ON 4°C	Donkey Anti-Rat IgG DyLight 550 (#NBP1-75652, NOVUSBIO) 1:200	Mice samples
Laminin	Rabbit IgG polyclonal Anti Laminin (#L9393, Sigma-Aldrich) 1:200 ON 4°C	Donkey Anti-Rabbit IgG DyLight 488 (#NBP1-75285, NOVUSBIO) 1:200	
Nuclei	/	Hoechst H33342 (#B2261, Sigma-Aldrich) 1:1000	
**Fiber Type**			
Fiber type I (MYH7)	Mouse IgG2b BA-D5-s (Developmental Studies Hybridoma Bank) 1:200 45min 37°C	Goat anti Mouse IgG2b Alexa Fluor 647 (#A21242, Thermo Fisher Scientific) 1:500	Rat samples (not use PFA)
Fiber type IIA (MYH2)	Mouse IgG1 SC-71-s (Developmental Studies Hybridoma Bank) 1:200 45min 37°C	Goat anti Mouse IgG1 Alexa Fluor 568 (#A21124, Thermo Fisher Scientific) 1:500	
Fiber type IIB (MYH4)	Mouse IgM BF-F3-s (Developmental Studies Hybridoma Bank) 1:50 45min 37°C	Goat anti Mouse IgM Alexa Fluor 488 (#A21042, Thermo Fisher Scientific) 1:500	
Laminin	Rabbit IgG polyclonal Anti Laminin (#ab11575, Abcam) 1:200 45min 37°C	Goat anti Rabbit IgG (H+L) Alexa Fluor 750 (#A21039, Thermo Fisher Scientific) 1:500	
Nuclei	/	Dapi in mouting medium (#ab104139, Abcam)	
**Fiber Intensity by ROI MB**			
Dystrophin	Mouse IgG2a MANDYS1(3B7)-s (Developmental Studies Hybridoma Bank) 1:250 ON 4°C	F(ab')2-Goat anti Mouse IgG (H+L) Alexa Fluor 647 (#A21237, Thermo Fisher Scientific) 1:500	Mice samples
Laminin	Rat IgG1 monoclonal Anti Laminin α2 (4H8-2) (#sc-59854, SantaCruz) 1:250 ON 4°C	Donkey anti Rat IgG (H+L) Alexa Fluor 488 (#A21208, Thermo Fisher Scientific) 1:500	
Dystrophin	Mouse IgG1 monoclonal Anti Dystrophin (#ab7164, Abcam) 1:200 ON 4°C	Goat anti Mouse IgG1 Alexa Fluor 568 (#A21124, Thermo Fisher Scientific) 1:500	Rat samples
Laminin	Rabbit IgG polyclonal Anti Laminin (#ab11575, Abcam) 1:200 ON 4°C	Goat anti Rabbit IgG (H+L) Alexa Fluor 488 (#A21206, Thermo Fisher Scientific) 1:500	
**Fiber Intensity by ROI F**			
Embryonic fiber type (MYH3)	Mouse IgG1 F1.652-s (Developmental Studies Hybridoma Bank) 1:250 ON 4°C	Donkey anti Mouse IgG (H+L) Alexa Fluor 488 (#A21202, Thermo Fisher Scientific) 1:500	Mice samples
Laminin	Rat IgG1 monoclonal Anti Laminin α2 (4H8-2) (#sc-59854, SantaCruz) 1:250 ON 4°C	Goat anti Rat IgG (H+L) Alexa Fluor 647 (#A21247, Thermo Fisher Scientific) 1:500	
**Capillaries/Fiber Type/Fiber morphology**			
Endothelial cells	Mouse IgG1 monoclonal Anti CD31 (#ab64543, Abcam) 1:200 1h 37°C (1st step)	Goat anti Mouse IgG1 Alexa Fluor 568 (#A21124, Thermo Fisher Scientific) 1:500 (1st step)	Rat samples (not use PFA)
Laminin	Rabbit IgG polyclonal Anti Laminin (#ab11575, Abcam) 1:200 1h 37°C (1st step)	Goat anti Rabbit IgG (H+L) Alexa Fluor 405 (#A31556, Thermo Fisher Scientific) 1:500 (1st step)	
Fiber type I (MYH7)	Mouse IgG2b BA-D5-s (Developmental Studies Hybridoma Bank) 1:200 45min 37°C (2nd step)	Goat anti Mouse IgG2b Alexa Fluor 647 (#A21242, Thermo Fisher Scientific) 1:500 (2nd step)	
Fiber type IIX (MYH1)	Mouse IgM 6H1-s (Developmental Studies Hybridoma Bank) 1:50 45min 37°C (2nd step)	Goat anti Mouse IgM Alexa Fluor 488 (#A21042, Thermo Fisher Scientific) 1:500 (2nd step)	

Supplementary references

1. Pertl C, Eblenkamp M, Pertl A, Pfeifer S, Wintermantel E, Lochmüller H, et al. A new web-based method for automated analysis of muscle histology. BMC Musculoskelet Disord. 2013; 14:26.

2. Kostrominova TY, Reiner DS, Haas RH, Ingermanson R, McDonough PM. Chapter Seven - Automated Methods for the Analysis of Skeletal Muscle Fiber Size and Metabolic Type. In: Jeon KW, éditeur. International Review of Cell and Molecular Biology [Internet]. Academic Press; 2013 [cité 18 oct 2022]. p. 275-332. (International Review of Cell and Molecular Biology; vol.306).

3. Beekman C, Sipkens JA, Testerink J, Giannakopoulos S, Kreuger D, van Deutekom JC, et al. A sensitive, reproducible and objective immunofluorescence analysis method of dystrophin in individual fibers in samples from patients with duchenne muscular dystrophy. PloS One. 2014;9(9):e107494.

4. Smith LR, Barton ER. SMASH - semi-automatic muscle analysis using segmentation of histology: a MATLAB application. Skelet Muscle. 2014;4:21.

5. Wen Y, Murach KA, Vechetti IJ, Fry CS, Vickery C, Peterson CA, et al. MyoVision: software for automated high-content analysis of skeletal muscle immunohistochemistry. J Appl Physiol Bethesda Md 1985. 2018;124(1):40-51.

6. Sardone V, Ellis M, Torelli S, Feng L, Chambers D, Eastwood D, et al. A novel high-throughput immunofluorescence analysis method for quantifying dystrophin intensity in entire transverse sections of Duchenne muscular dystrophy muscle biopsy samples. PloS One. 2018;13(3):e0194540.

7. Mayeuf-Louchart A, Hardy D, Thorel Q, Roux P, Gueniot L, Briand D, et al. MuscleJ: a high-content analysis method to study skeletal muscle with a new Fiji tool. Skelet Muscle. 2018;8(1):25.

8. Lau YS, Xu L, Gao Y, Han R. Automated muscle histopathology analysis using CellProfiler. Skelet Muscle. 2018;8(1):32.

9. Desgeorges T, Liot S, Lyon S, Bouvière J, Kemmel A, Trignol A, et al. Open-CSAM, a new tool for semi-automated analysis of myofiber cross-sectional area in regenerating adult skeletal muscle. Skelet Muscle. 2019;9(1):2.

10. Aeffner F, Faelan C, Moore SA, Moody A, Black JC, Charleston JS, et al. Validation of a Muscle-Specific Tissue Image Analysis Tool for Quantitative Assessment of Dystrophin Staining in Frozen Muscle Biopsies. Arch Pathol Lab Med. 2019;143(2):197-205.

11. Reyes-Fernandez PC, Periou B, Decrouy X, Relaix F, Authier FJ. Automated image-analysis method for the quantification of fiber morphometry and fiber type population in human skeletal muscle. Skelet Muscle. 2019;9(1):15.

12. Kastenschmidt JM, Ellefsen KL, Mannaa AH, Giebel JJ, Yahia R, Ayer RE, et al. QuantiMus: A Machine Learning-Based Approach for High Precision Analysis of Skeletal Muscle Morphology. Front Physiol. 2019; 10:1416.

13. Rettig A, Haase T, Pletnyov A, Kohl B, Ertel W, von Kleist M, et al. SLCV-a supervised learning-computer vision combined strategy for automated muscle fibre detection in cross-sectional images. PeerJ. 2019;7:e7053.

14. Sanz G, Martínez-Aranda LM, Tesch PA, Fernandez-Gonzalo R, Lundberg TR. Muscle2View, a CellProfiler pipeline for detection of the capillary-to-muscle fiber interface and high-content quantification of fiber type-specific histology. J Appl Physiol Bethesda Md 1985. 2019;127(6):1698-709.

15. Encarnacion-Rivera L, Foltz S, Hartzell HC, Choo H. Myosoft: An automated muscle histology analysis tool using machine learning algorithm utilizing FIJI/ImageJ software. PloS One. 2020; 15(3):e0229041.

16. Scaglioni D, Ellis M, Catapano F, Torelli S, Chambers D, Feng L, et al. A high-throughput digital script for multiplexed immunofluorescent analysis and quantification of sarcolemmal and sarcomeric proteins in muscular dystrophies. Acta Neuropathol Commun. 2020;8(1):53.

17. Babcock LW, Hanna AD, Agha NH, Hamilton SL. MyoSight-semi-automated image analysis of skeletal muscle cross sections. Skelet Muscle. 2020;10(1):33.

18. Bonilla HJ, Messi ML, Sadieva KA, Hamilton CA, Buchman AS, Delbono O. Semiautomatic morphometric analysis of skeletal muscle obtained by needle biopsy in older adults. GeroScience. 2020;42(6):1431-43.

19. Stevens CR, Berenson J, Sledziona M, Moore TP, Dong L, Cheetham J. Approach for semi-automated measurement of fiber diameter in murine and canine skeletal muscle. PloS One. 2020;15(12):e0243163.

20. Bindellini D, Voortman LM, Olie CS, van Putten M, van den Akker E, Raz V. Discovering fiber type architecture over the entire muscle using data-driven analysis. Cytometry A. 2021;99(12):1240-9.

21. Waisman A, Norris AM, Elías Costa M, Kopinke D. Automatic and unbiased segmentation and quantification of myofibers in skeletal muscle. Sci Rep. 2021;11(1):11793.

22. Rahmati M, Rashno A. Automated image segmentation method to analyse skeletal muscle cross section in exercise-induced regenerating myofibers. Sci Rep. 2021;11(1):21327.

23. Vetter TA, Nicolau S, Bradley AJ, Frair EC, Flanigan KM. Automated immunofluorescence analysis for sensitive and precise dystrophin quantification in muscle biopsies. Neuropathol Appl Neurobiol. 2022;48(3):e12785.

24. Laghi V, Ricci V, De Santa F, Torcinaro A. A User-Friendly Approach for Routine Histopathological and Morphometric Analysis of Skeletal Muscle Using CellProfiler Software. Diagnostics. 2022;12(3):561.

25. Viggars MR, Wen Y, Peterson CA, Jarvis JC. Automated cross-sectional analysis of trained, severely atrophied, and recovering rat skeletal muscles using MyoVision 2.0. J Appl Physiol Bethesda Md 1985. 2022;132(3):593-610.
